# Association between physician's case volume in prehospital advanced trauma care and 30-day mortality: A registry-based analysis of 4,032 patients

**DOI:** 10.1097/TA.0000000000003777

**Published:** 2022-09-08

**Authors:** Anssi Saviluoto, Jukka Pappinen, Hetti Kirves, Lasse Raatiniemi, Jouni Nurmi

**Affiliations:** From the University of Eastern Finland, Faculty of Health Sciences (A.S., J.P.), Kuopio; Emergency Medicine and Services (H.K., J.N.), Helsinki University Hospital; Emergency Medicine (H.K., J.N.), University of Helsinki, Helsinki; and Centre for Prehospital Emergency Care (L.R.), Oulu University Hospital, Oulu, Finland.

**Keywords:** Trauma, air ambulances, emergency medical services, critical care, quality

## Abstract

Seriously injured patients may benefit from prehospital interventions provided by a critical care physician. The relationship between case volume and outcome has been established in trauma teams in hospitals, as well as in prehospital advanced airway management. In this study, we aimed to assess if a volume-outcome relationship exists in prehospital advanced trauma care.

**METHODS:**

We performed a retrospective cohort study using the national helicopter emergency medical services database, including trauma patients escorted from scene to hospital by a helicopter emergency medical services physician during January 1, 2013, to August 31, 2019. In addition, similar cases during 2012 were used to determine case volumes. We performed a multivariate logistic regression analysis, with 30-day mortality as the outcome. Age, sex, Glasgow Coma Scale, shock index, mechanism of injury, time interval from alarm to the patient and duration of transport, level of receiving hospital, and physician's trauma case volume were used as covariates. On-scene times, interventions performed, and status at hospital arrival were assessed in patients who were grouped according to physician's case volume.

**RESULTS:**

In total, 4,032 escorted trauma patients were included in the study. The median age was 40.2 (22.9–59.3) years, and 3,032 (75.2%) were male. Within 30 days, 498 (13.2%) of these patients had died. In the highest case volume group, advanced interventions were performed more often, and patients were less often hypotensive at handover. Data for multivariate analysis were available for 3,167 (78.5%) of the patients. Higher case volume was independently associated with lower mortality (odds ratio, 0.59; 95% confidence interval, 0.38–0.89).

**CONCLUSION:**

When a prehospital physician's case volume is higher in high-risk prehospital trauma, this seems to be associated with more active practice patterns and significantly lower 30-day mortality. The quality of prehospital critical care could be increased by ensuring sufficient case volume for the providers of such care.

**LEVEL OF EVIDENCE:**

Prognostic and Epidemiologic; Level III.

## Abstract 2

New study suggests that organizing advanced prehospital trauma care in a way that ensures physicians encounter trauma patients frequently might improve patient survival.

## Graphical abstract

**Figure:**
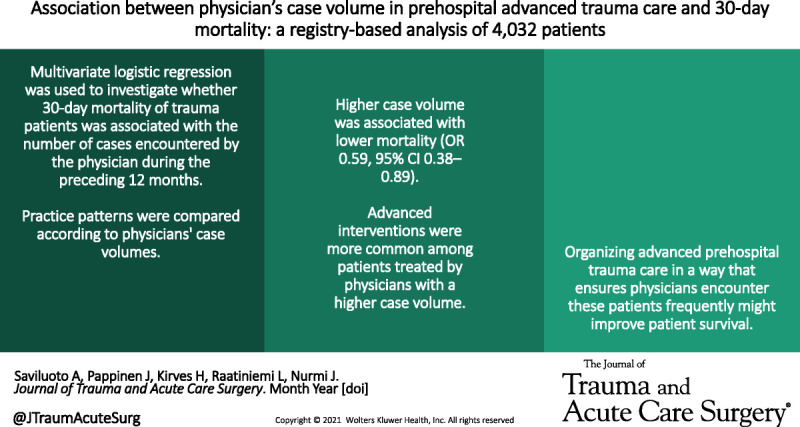

Reducing the delay to definitive care decreases the mortality rate in major trauma.^1^ However, as prehospital care has advanced, many urgent interventions have become available in the prehospital setting. Many modern studies have contested the causal relationship between on-scene time and survival, especially when controlling for lifesaving procedures provided on-scene.^1,2^ This has led to discussion on which interventions should be initiated during the prehospital phase. Critically injured patients may benefit from on-scene interventions when performed by providers well versed in these situations.

Most ground-based units encounter seriously injured patients rarely. Helicopter emergency medical services (HEMSs) may be used cover larger populations and thus enable specialized prehospital trauma teams to gain more exposure to these situations.^3^ The results from studies addressing the effectiveness of HEMSs or prehospital critical care in trauma patients are inconsistent.^4–6^ Potential reasons for mixed results include differences in mechanisms of injury (penetrating vs. blunt), durations of transport, in-hospital treatment, and also differences in the composition and experience of the HEMSs crew.

Association between case volume and outcome has been recognized in many specialties and health care services, both on the provider and hospital levels.^7–9^ This has led to the centralization of treatment at larger-volume centers for rare and challenging conditions. However, the literature on the relationship between case volume and outcome in prehospital critical care is limited. Recently, an association between the case volume of HEMSs physicians and mortality after prehospital anesthesia was found.^10^ For patients with major trauma, dedicated teams are allocated in the emergency department, and the most severe cases are transported to dedicated trauma centers.^11,12^ Trauma centers with higher case volumes have been shown to achieve better outcomes, which has led to minimum volume standards being implemented in many countries.^13^

Based on observations on the volume-outcome relationship in prehospital anesthesia and hospital-based trauma care, we hypothesized that a similar relationship also exists in advanced prehospital trauma care provided by HEMSs physicians. The current study aimed to assess if HEMSs physician's case volume in prehospital trauma was associated with 30-day mortality of patients.

## PATIENTS AND METHODS

### Design

We performed a retrospective cohort study based on the national HEMSs quality database to assess the association between the number of high-risk prehospital trauma patients treated by a HEMSs physician in the preceding 12 months (i.e., the case volume) and 30-day mortality after severe trauma. The study protocol was approved by an ethical committee, and access to the data was granted by all hospitals responsible for HEMSs (200/2019 2.7.2019, 280/2019 9.7.2019, J30/19 4.8.2019, 32/2019 22.8.2019, RPL 102/2019 22.8.2019, RTL-R19580 2.9.2019), the Finnish Institute for Health and Welfare (THL/2231/5.05.00/2019), and the Digital and Population Data Services Agency (VRK/5613/2019-3). The study did not affect patient treatment, and therefore, patient consent was not required nor acquired. The Strengthening the Reporting of Observational Studies in Epidemiology statement is followed in reporting this study (Supplemental Digital Content, Supplementary Table 1, http://links.lww.com/TA/C676).^14^

### Setting

The Finnish HEMSs consist of five physician-staffed helicopter units located near the university hospitals and one unit staffed by advanced paramedics serving the sparsely populated northern part of the country. Each unit operates by helicopter or car depending on which is more practical because of weather conditions or distance. In Finland, HEMSs are part of a publicly funded health care system and work closely with other emergency services. Helicopter emergency medical services units are dispatched by the emergency communication centers simultaneously with other units based on predefined criteria, including major trauma, out-of-hospital cardiac arrest, and unconsciousness. In addition, ambulance personnel can request HEMSs dispatch. In the country, HEMSs respond only to primary missions, with some rare exceptions. Finnish HEMSs have been recently described in detail.^15^

Most HEMSs physicians during the study period were senior anesthesiologists, of which a quarter consisted of anesthesiology residents in their final year of specialization, and a few physicians were final-year residents or consultants in other specialties. Only a minority work full time in HEMSs. During the study period, most did regular shifts in-hospital (e.g., as anesthesiologists in intensive care units and operating rooms or clinicians in the emergency department). No uniform requirements for physician training or experience were in place during the study period. The frequency of prehospital work varies between physicians. We have described the range of missions and variation in exposure between providers in previous papers.^15,16^

### Participants

We included trauma patients who had been encountered and escorted by a HEMSs physician between January 1, 2013, and August 31, 2019. Patients encountered during 2012 were used to determine physicians' case volumes but were not included in analyses. Only patients escorted or transported by HEMSs were chosen, as the decision not to escort patients usually indicates only minor trauma that is unlikely to need advanced-level care; conversely, patients escorted by HEMSs usually have signs of severe injury.^17^ Patients who deceased during prehospital care and patients with missing outcome data were excluded from the analysis.

### Variables

Trauma case volume was defined as the number of patients fulfilling the inclusion criteria who were encountered by a HEMSs physician during the 12 months preceding the date of each included case. Consequently, physician's case volume was calculated per patient, and therefore, the case volume of any single physician may vary depending how often they encountered trauma patients. The primary outcome was all-cause 30-day mortality. The secondary outcome was quality of physiological stabilization of the patient, defined as the change in shock index (SI) during the care provided by the physician. Shock index is defined as heart rate divided by systolic blood pressure.^18^ We calculated the change in SI during prehospital care only for patients for whom it was elevated when encountered by HEMSs to estimate the effectiveness of hemodynamic stabilization.

To control for confounding factors, multiple other variables were included. The severity of physiological disturbance was measured by SI, calculated from the values measured at the time of patient contact by HEMSs crew. Similarly, the Glasgow Coma Scale (GCS) score at the time of patient contact was used to control for the level of consciousness. For patients sedated before being encountered by HEMSs (n = 51), we used the GCS at first emergency medical services contact (n = 6); if not available, we assumed sedated patients to have a GCS score of 3. We classified the mechanism of injury into five categories according to the dispatch code assigned by the emergency response center: fall, fall from height, traffic accident, violence, and other. We also classify the main type of injury as blunt or penetrating according to the HEMSs physician. In addition, age, sex, time from alarm to patient contact, duration of transport, and whether the patient was transported directly to a university hospital were included in the analyses.

Transport time was defined as the beginning of transport by helicopter or ambulance to arrival at the hospital. Some patients needed secondary transport with an ambulance from the helicopter landing site to the hospital, as not all hospitals have a dedicated helipad. These secondary transports were included in the transport time. On-scene time was determined as the time from HEMSs arriving at the scene to the beginning of transport.

We reported the severity of anatomical injuries by calculating diagnosis-specific survival probabilities from hospital diagnoses using the international pool for the *International Classification of Diseases, Tenth Revision*–based Injury Severity Score (ICISS).^19^

### Data Sources

Data collection was based on the national HEMSs quality registry. Data on every HEMSs mission in the country have been entered into the database since 2012. The collected variables include operational (e.g., timestamps, crew members, the vehicle used) and detailed clinical data. The recommendations for data collection from physician-provided prehospital care and advanced airway management are followed.^15^

Based on the personal identification number, issued to every resident in Finland, diagnoses and mortality data were linked with cases in the HEMSs quality register. Data from diagnoses during the hospital stay following the incident were collected from the national hospital discharge registry. Up to five primary diagnoses were attained for each patient. Data entry into this registry are mandatory for all hospitals in the country. Mortality data were acquired from the Digital and Population Data Services Agency, responsible for the population registry in Finland, which holds data on all permanent and temporary residents in the country.

### Statistical Methods

For the primary analysis, we performed a multivariate logistic regression analysis, using 30-day all-cause mortality as the outcome. We included age, sex, GCS, SI, mechanism of injury, time interval from alarm to the patient and duration of transport, whether the patient was transported directly to a university hospital, and physician's prehospital trauma case volume as covariates. In this multivariate analysis, we used a base 10 logarithm of the number of trauma cases in the preceding 12 months because of the presumably different effect of a single additional case in a low versus a high case volume. The SI was categorized into five categories based on the earlier observation of a U-shape association between SI and mortality.^20^ The ICISS was not included in the model because of the large amount of missing data. However, a multivariate logistic regression analysis including the ICISS was performed as a sensitivity analysis. We also conducted a sensitivity analysis excluding patients with a GCS score of above 12 and another, where we excluded all patients who did not live through the day following the injury. The results of the model are reported as odds ratios (ORs).

As a secondary analysis to assess differences in practice patterns by case volume, the patients were divided into three groups according to the treating physician's prehospital trauma case volume: low (0–10 cases per 12 months), intermediate (11–20 cases per 12 months), and high (>20 cases per 12 months). The categorization was done before the analyses by visually looking at the distribution of patients according to case volume to define three separate categories. Categories are purposefully unequal in size as the highest case volumes are difficult to achieve but demark the cases of interest. We compared medical management between the groups, specifically the proportions of patients receiving advanced interventions, on-scene times, and proportions of hypotensive and hypoxic patients at hospital arrival, as well as the proportion of GCS score of ≤9 at hospital arrival without advanced airway. Furthermore, to assess hemodynamic stabilization, the change in SI during prehospital care was compared between groups.

All proportions are reported as percentages with 95% confidence intervals (CIs). Continuous variables are reported as medians (25th–75th percentile). *p* Values for continuous values were calculated using the Kruskal-Wallis test for independent samples. For proportions, we used the Pearson χ^2^ test.

Missing data were excluded from the analysis. The proportion of missing data for each variable is reported in an online supplement (Supplemental Digital Content, Supplementary Table 2, http://links.lww.com/TA/C677). The study size was not determined by power calculations, as we instead used all the available data. All statistical analyses were done using SPSS Statistics for Mac, version 27 (IBM Corp., Armonk, NY).

## RESULTS

**Figure 1:**
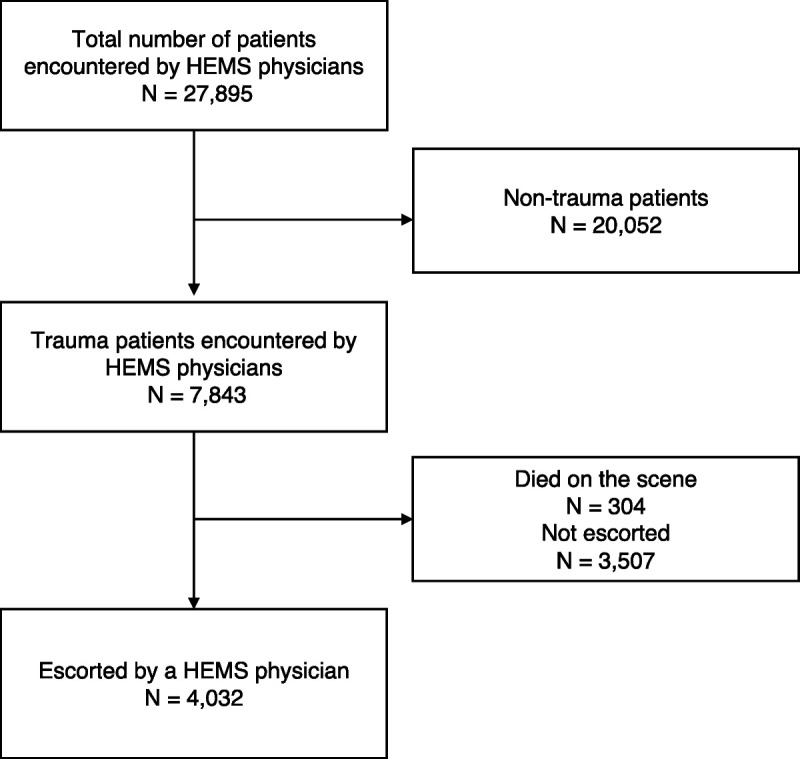
Patient selection flow chart.

**Figure 2:**
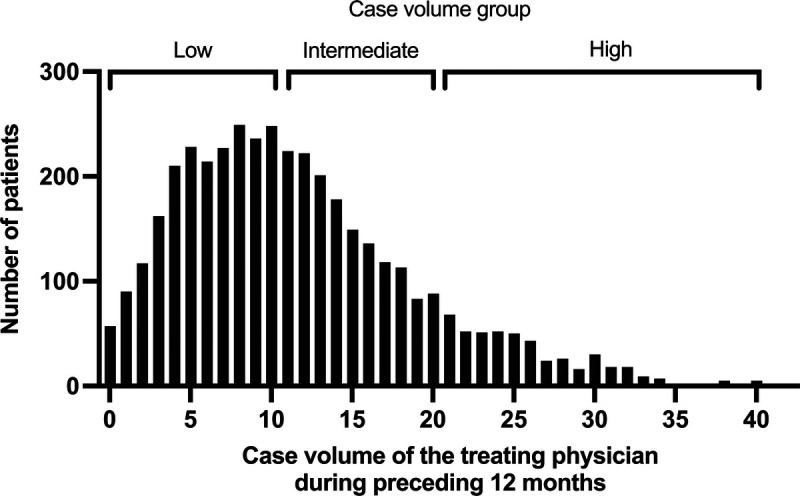
The number of patients according to case volume of the treating physician. The case volume was defined as the number of patients fulfilling the inclusion criteria and encountered by the physician during the preceding 12 months. The patients were categorized according to case volume into three groups.

Helicopter emergency medical services physicians escorted 4,032 trauma patients during the study period, and all of them were included in the analyses (Fig. 1). The median age of patients was 40.2 (22.9–59.3) years, and 3,032 (75.2%) of them were male. A total of 671 patients (16.6%) were transported by helicopter, with the remainder escorted in an ambulance. The distribution of patients according to the treating physician's previous case volume is presented in Figure 2. The numbers of patients treated by physicians with low, intermediate, and high case volumes were 2,038 (50.5%), 1,512 (37.5%), and 482 (12.0%), respectively. The characteristics and a comparison between the groups are presented in Table 1. Mortality data were available for 3,786 (93.9%) of the patients. Overall, 498 patients (13.2%) died within 30 days.

**TABLE 1:** Characteristics of the Trauma Patients Encountered and Escorted or Transported by HEMSs Physician by the Number of Trauma Cases of the Physician During Preceding 12 Months

	0–10 Cases n = 2,038		11–20 Cases n = 1,512		>20 Cases n = 482		Missing	
Age, y	41	(22–60)	38	(23–58)	45	(27–62)	7	(0.2)
Sex, male	1,503	(74)	1,158	(77)	371	(77)	17	(0.4)
Mechanism of trauma based on dispatch code (%)							0	(0)
Fall on the same level	192	(9.4)	137	(9.1)	52	(11)		
Fall from height	208	(10)	174	(12)	63	(13)		
Traffic incident	933	(46)	661	(44)	200	(42)		
Violence	238	(12)	220	(15)	58	(12)		
Other	467	(23)	320	(21)	109	(23)		
Dominating type of injury							1	(0.02)
Blunt	1,742	(85)	1,249	(83)	407	(84)		
Penetrating	268	(13)	244	(16)	73	(15)		
Other	27	(1)	19	(1)	2	(0.4)		
Physiological status when encountered								
Heart rate, min^−1^	90	(80–108)	91	(79–110)	90	(76–108)	192	(4.8)
Systolic blood pressure, mm Hg	128	(110–146)	128	(110–148)	130	(110–148)	409	(10.1)
SI	0.71	(0.57–0.88)	0.72	(0.57–0.89)	0.69	(0.56–0.86)	446	(11.1)
GCS score	14	(6–15)	14	(8–15)	14	(7–15)	41	(1.0)
Oxygen saturation	97	(94–99)	97	(94–99)	97	(94–99)	405	(10.0)
ICISS	0.90	(0.76–0.97)	0.91	(0.79–0.98)	0.90	(0.77–0.97)	970	(24.1)
Time from alarm to HEMSs on-scene, min	26	(17–40)	24	(16–36)	26	(17–37)	0	(0)
Transport time to hospital	29	(17–42)	28	(16–40)	29	(17–40)	219	(5.4)

Categorical data are presented as n (%), and continuous data as median (25–75 percentiles).

The practice patterns differed according to the case volume of the physician (Table 2). The patients in the highest case volume group received actual values shown in Table 2 pleural decompression more often.

**TABLE 2:** Prehospital Management and Outcomes of Trauma Patients According to physician's Trauma Case Volume During the Past 12 Months

	<10 Cases n = 2,038		10–20 Cases n = 1,512		>20 Cases n = 482		*p*
On-scene time, min	16	(6–28)	16	(8–26)	17	(9–27)	0.428*
Transported by helicopter	338	(16)	247	(16)	86	(18)	0.738**
Advanced airway management							0.155**
Mask ventilation	23	(1.1)	9	(0.6)	10	(2.1)	
Supraglottic	9	(0.4)	5	(0.3)	0	(0)	
Intubation	672	(33)	488	(32)	164	(34)	
Surgical airway	1	(0.0)	3	(0.2)	1	(0.2)	
Drug facilitated airway management	683	(34)	495	(33)	157	(33)	0.858**
Pleural decompression	51	(2.5)	51	(3.4)	28	(5.1)	0.001**
Hemostatic procedure	192	(9.4)	180	(12)	51	(11)	0.058**
Use of vasoactives	411	(20)	280	(19)	113	(23)	0.058**

Categorical data are presented as n (%), and continuous data as median (25–75 percentiles).

*Kruskal-Wallis test.

**Pearson χ^2^ test.

The primary and secondary outcomes of the patient groups are presented in Table 3. Patients treated by physicians with higher case volumes were more often intubated if GCS score was less or equal to 9 and were less often hypotensive at handover. Conversely, the rate of hypoxia at handover was similar between the groups, and no difference was seen in SI improvement among those with elevated SI at encounter. Unadjusted 30-day mortality was lowest in the intermediate case volume group, whereas 1-year mortality did not differ.

**TABLE 3:** Outcome According to Number of Prehospital Trauma Cases Encountered by HEMSs Physician in Preceding 12 Months

	<10 Cases n = 2,038		10–20 Cases n = 1,512		>20 Cases n = 482		*p*
Hypoxic at handover to hospital	18	(1)	30	(2.2)	9	(2)	0.683*
Hypotensive at handover to hospital	96	(5.6)	78	(5.9)	20	(4.8)	0.025*
Patients with GCS ≤9 at hospital without advanced airway**	124/740	(17)	66/537	(12)	20/176	(11)	0.038*
Mortality 30 d	275	(14)	160	(11)	63	(14)	0.028*
Mortality 1 y	320	(19)	198	(15)	66	(16)	0.051*
Median change in SI†	−0.077	(−0.21 to 0.026)	−0.091	(−0.21 to 0.03)	−0.11	(−0.24 to 0.00)	0.375‡

*Pearson χ^2^ test.

**Advanced airway defined as surgical airway or tracheal intubation.

†Shock index calculated from patients with elevated SI at time of HEMSs encounter.

‡Kruskal-Wallis test for independent samples, n = 948, 703, and 212 for groups of <10, 10 to 20, and >20 cases, respectively.

Categorical data are presented as n (%) and continuous data as median (25–75 percentiles).

**Figure 3:**
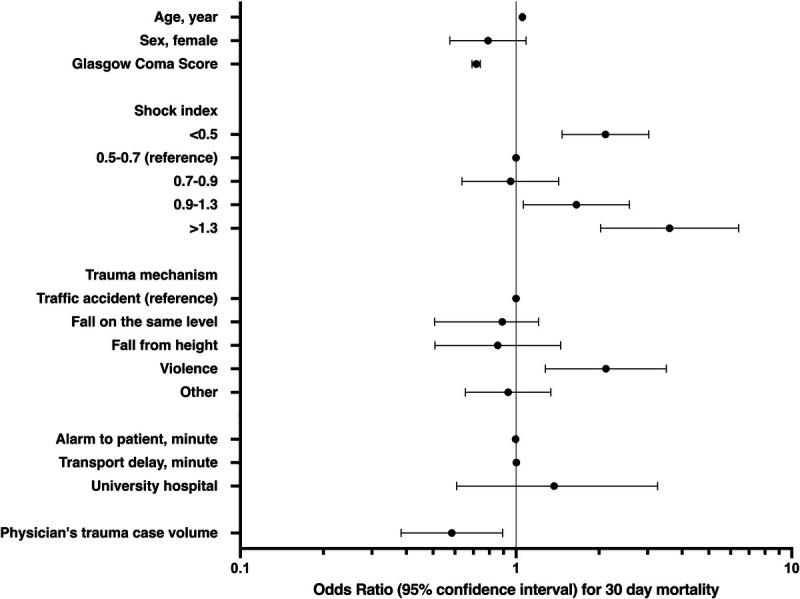
Multivariate logistic regression analysis of 30-day mortality in trauma patients escorted by HEMSs physicians. A physician's trauma case volume is represented by the logarithm of trauma patients encountered and escorted by the physician during the preceding 12 months.

Sufficient data for multivariate analysis were available for 3,167 (78.5%) of the patients. In this model, higher case volume was independently associated with lower mortality (OR, 0.59; 95% CI, 0.38–0.89) (Fig. 3). In the model with ICISS included as a covariant (n = 2,526), CIs were wider and crossed 1.0 (OR, 0.68; 95% CI, 0.41–1.13) (Supplemental Digital Content, Supplementary Fig. 1, http://links.lww.com/TA/C678). This was also the case in the other sensitivity analyses. Excluding patients who did not survive the day following the HEMSs encounter left 2,935 patients with all necessary variables recorded (OR, 0.63; 95% CI, 0.34–1.00). Excluding patients with GCS score of >12 at time of HEMSs encounter resulted in 1,264 patients in the multivariate analysis (OR, 0.65; 95% CI, 0.41–1.04).

## DISCUSSION

This study demonstrates a strong association between the case volume of an advanced prehospital trauma care provider and 30-day mortality after severe trauma. The more active practice patterns of the high-volume providers, namely, more frequent airway management of patients with a decreased level of consciousness and pleural decompression, support the hypothesis of a causal relationship. While unadjusted mortality did not differ according to physician case volume, only a small proportion of patients died overall. Of these, a large proportion would have survived or perished regardless. Therefore, the only way to demonstrate this effect is to control prognostic factors on a patient-by-patient basis to identify those individuals whose survival might have been impacted by physician case volume.

The concept of a volume-outcome relationship is well recognized in many specialties and settings, including in-hospital acute trauma care. A systematic review and meta-analysis by Sewalt et al.^13^ identified 18 studies evaluating the relationship between hospital or surgeon volume and health outcomes among seriously injured patients. Seventy-two percent of these studies reported larger case volumes to be associated with improved outcomes. The meta-analysis showed an annual volume of more than 240 admitted seriously injured patients of the hospital associating with lower risk of mortality (OR, 0.85; 95% CI, 0.76–0.94).^13^

There are few studies exploring the volume-outcome relationship in prehospital critical care. A survey of nonphysician emergency medical services personnel in Northern Finland reported a mean annual frequency of 1.1 drug-assisted intubations among providers licensed to perform the procedure.^21^ In our previous studies, we found the median to be 11 per year among Finnish HEMSs physicians and a higher annual number to be associated lower mortality.^10,16^ However, the volume-outcome relationship in prehospital critical care does not seem to be universal, as more active treatment for patients resuscitated from out-of-hospital cardiac arrest by physicians with high case volumes did not lead to higher survival rates.^22^ To better understand the sorts of prehospital emergencies benefiting from centralization warrants studies like this one.

The key components of prehospital advanced trauma care include control of hemorrhage, airway management, pleural decompression, high-ratio transfusion for volume replacement and prevention of trauma-induced coagulopathy, triage decisions, and rapid evacuation to the most appropriate hospital.^23^ Anesthesia and procedural sedation are frequently deployed.^24^ Patients with traumatic brain injury may benefit from high-quality anesthesia and controlled ventilation.^25^ To perform the most appropriate interventions, the prehospital trauma provider has to make decisions based on examining the patient and understanding the mechanism of injury. The importance of clinical judgment is emphasized by the limited availability of additional diagnostic equipment, as ultrasound is practically the only imaging modality readily available in a prehospital environment. Taking all this together, the skill set required for advanced prehospital trauma management may be seen as a combination of the skill sets of a trauma surgeon, trauma team leader, and trauma anesthesiologist. In addition, the changing and sometimes insecure working environment may increase the complexity of advanced prehospital trauma care. These factors make prehospital advanced trauma care its own entity. Thus, the skill transfer from hospital-based work may not be sufficient to provide high expertise, and therefore, frequent exposure in the prehospital setting is needed.

Organizational interventions to increase case volume may improve the quality of care in prehospital critical care. These interventions include, for example, limiting the number of providers, changing the composition of the work, and organizing rotation between low- and high-volume units. However, to ascertain this effect, prospective quality improvement studies are needed. Standard operating procedures, simulation training, and persistent clinical governance might compensate for lower case volumes to some degree.

The case volume of providers could be one explanatory factor for the inconsistent results between studies in prehospital critical care.^26^ For example, for prehospital intubation, the annual case number per physician varies from 5 to 20, the higher volumes including only rapid sequence intubations, as reported by two Nordic services staffed by anesthesiologists.^27,28^

It must be noted that this study did not analyze the volume of nontrauma HEMSs missions. It is likely that providers who achieved the largest volumes of trauma cases also saw more medical emergencies. Many skills may transfer over, and our previous paper suggests that overall case volume might be important to perform optimally in the prehospital setting.^16^ It is however essential to explore specific patient categories to identify groups that may benefit from centralized prehospital care, as this effect does not seem to universally apply to all emergencies.^22^ Findings like the one presented in this paper direct which missions could be forwarded to specialized teams. We strongly recommend that, in addition to the provider's educational level and experience, the case volume of the providers is reported in studies concerning prehospital critical care.

The strengths of the current study include the robust database covering the whole national HEMSs. Our findings demonstrate the importance of regular routine in prehospital trauma care, and we believe that all services should aim to monitor case volume of HEMSs physicians and teams. We suggest that case volume should have a central role in quality improvement efforts.

The present study has several limitations. First, because there is no nationwide trauma registry in Finland, we were unable to include injury severity scoring. However, we report physiological parameters and included only the patients escorted by HEMSs. Second, we were unable to document case volume in the hospital for individual physicians or the experience and education in trauma care such as participation in trauma courses or systematic simulation training. Third, some HEMSs units begun to stock blood products during the study period, but use of blood products was not recorded in the database. Therefore, we cannot say if this practice differed according to physician case volume. Finally, even though the groups of patients had similar baseline characteristics and many confounders were addressed by the multivariate analysis, any case selection bias by the providers cannot by fully excluded.

## CONCLUSION

A higher case volume for a prehospital physician in high-risk prehospital trauma seems to be associated with a more active practice pattern and significantly lower 30-day mortality.

## Supplementary Material
