# Supplementary material for: Association between physician's case volume in prehospital advanced trauma care and 30-day mortality: A registry-based analysis of 4,032 patients
Source: J Trauma Acute Care Surg. 2022 Sep 8;94(3):425–32. doi: 10.1097/TA.0000000000003777 (PMC9940832; doi:10.1097/TA.0000000000003777)
Supplement: Supplementary file 2 [file jt-94-425-s002.docx]

Supplement 1

Missing data

Reported as *n* (%). Total n = 4,032

|  | Cannot be measured | | Was not measured or recorded | | System missing | |
| --- | --- | --- | --- | --- | --- | --- |
| Patient characteristics |  |  |  |  |  |  |
| Age | 0 | 0 | 0 | 0 | 7 | 0.2 |
| Sex | 0 | 0 | 0 | 0 | 17 | 0.4 |
| Mechanism of trauma based on dispatch code | 0 | 0 | 0 | 0 | 0 | 0 |
| Dominating type of injury | 0 | 0 | 0 | 0 | 1 | 0.02 |
| Physiological status when encountered |  |  |  |  |  |  |
| Heart rate at encounter | 0 | 0 | 0 | 0 | 192 | 4.8 |
| Heart rate at hospital | 0 | 0 | 0 | 0 | 331 | 8.2 |
| Systolic blood pressure at time of patient encounter | 0 | 0 | 0 | 0 | 409 | 10.1 |
| Shock index | 0 | 0 | 0 | 0 | 446 | 11.1 |
| Glasgow coma score at time of patient encounter | 0 | 0 | 0 | 0 | 41 | (1.0) |
| Glasgow coma score at hospital | 0 | 0 | 0 | 0 | 225 | (5.6) |
| Oxygen saturation at time of patient encounter | 238 | 5.9 | 67 | 1,7 | 100 | 1.5 |
| Systolic blood pressure at hospital | 108 | 2.7 | 0 | 0 | 436 | 10.8 |
| Oxygen saturation at hospital | 175 | 4.3 | 57 | 1.4 | 227 | 5.6 |
| ICISS | 0 | 0 | 0 | 0 | 970 | 24.1 |
| Time from alarm to HEMS on-scene | 0 | 0 | 0 | 0 | 0 | 0 |
| Transport time to hospital | 0 | 0 | 0 | 0 | 219 | 5.4 |
| On-scene time | 0 | 0 | 0 | 0 | 3 | 0.1 |
| Advanced airway management | 0 | 0 | 0 | 0 | 22 | 0.5 |
| Anesthetics used in induction | 0 | 0 | 0 | 0 | 0 | 0 |
| CPR | 0 | 0 | 0 | 0 | 0 | 0 |
| Pleural decompression | 0 | 0 | 0 | 0 | 0 | 0 |
| Hemostatic procedure | 0 | 0 | 0 | 0 | 0 | 0 |
| Use of vasoactives | 0 | 0 | 0 | 0 | 0 | 0 |
| Hospital level | 0 | 0 | 0 | 0 | 0 | 0 |
| Mortality 30 days | 0 | 0 | 0 | 0 | 246 | 6.1 |
| Mortality 1 year | 0 | 0 | 0 | 0 | 591 | 14.7 |

CPR = cardiopulmonary resuscitation
